# Post-Release Monitoring of Western Grey Kangaroos (*Macropus* *fuliginosus*) Relocated from an Urban Development Site

**DOI:** 10.3390/ani10101914

**Published:** 2020-10-19

**Authors:** Mark Cowan, Mark Blythman, John Angus, Lesley Gibson

**Affiliations:** 1 Biodiversity and Conservation Science, Department of Biodiversity, Conservation and Attractions, Wildlife Research Centre, Woodvale, WA 6026, Australia; mark.blythman@dbca.wa.gov.au (M.B.); john.angus@dbca.wa.gov.au (J.A.); 2 Biodiversity and Conservation Science, Department of Biodiversity, Conservation and Attractions, Kensington, WA 6151, Australia; lesley.gibson@dbca.wa.gov.au

**Keywords:** kangaroo management, relocation, human-wildlife conflict, GPS telemetry, urbanisation

## Abstract

**Simple Summary:**

As a result of urban development, 122 western grey kangaroos (*Macropus fuliginosus*) were relocated from the outskirts of Perth, Western Australia, to a nearby forest. Tracking collars were fitted to 67 of the kangaroos to monitor survival rates and movement patterns over 12 months. Spotlighting and camera traps were used as a secondary monitoring technique particularly for those kangaroos without collars. The survival rate of kangaroos was poor, with an estimated 80% dying within the first month following relocation and only six collared kangaroos surviving for up to 12 months. This result implicates stress associated with the capture, handling, and transport of animals as the likely cause. The unexpected rapid rate of mortality emphasises the importance of minimising stress when undertaking animal relocations.

**Abstract:**

The expansion of urban areas and associated clearing of habitat can have severe consequences for native wildlife. One option for managing wildlife in these situations is to relocate them. While there is a general perception that relocation is humane, transparency of outcomes is lacking. Here, we document the outcome of 122 western grey kangaroos (*Macropus fuliginosus*) relocated from an urban development site on the edge of Perth, Western Australia. Global Positioning System (GPS) or Very High Frequency (VHF) collars were fitted to 67 kangaroos, and their survival and movement were monitored over 12 months using telemetry, camera traps and spotlighting. Only six collared animals survived for the duration of the study with most dying within a week of the relocation, indicating stress associated with capture as the likely cause. By the completion of the study, 111 kangaroos were predicted to have died based on the proportion of individuals known to have died. Movement patterns of surviving GPS collared kangaroos changed over time from largely exploratory forays, to more repeated movements between focus areas within home ranges. The poor outcome here raises concerns around the viability of relocating a relatively large number of kangaroos as a management option. It also highlights the need for careful planning to limit the stress associated with capture and transport if relocations are to be used for managing kangaroos in urban areas.

## 1. Introduction

The relocation of native fauna out of the path of development projects, sometimes termed mitigation translocation, has dramatically increased in recent years, most likely in response to the rapid pace of urban development [1]. As a wildlife management tool, relocation for mitigation purposes differs in motive from conservation-driven translocations as these relocations are more concerned about the removal of individuals out of harm’s way rather than establishing populations to improve conservation status [2,3]. Despite the rise in number of relocations, there has been a lack of transparency in terms of the outcomes of these relocations [1,4], though those that have been documented generally indicate poor success [1,5,6]. A further complication common to relocations for mitigation purposes is that animals are often moved to an area where conspecifics occur without an understanding of the impacts on either the relocated or resident population [4].

In Australia, the continued development of native bushland and semi-rural areas on the fringes of metropolitan areas and major regional centres has highlighted the need for the considered management of wildlife in these areas [7,8,9,10,11,12]. As land is cleared or re-zoned for development, animals often become stranded and concentrated in the last remaining fragments [12,13]. Where development projects involve the clearing of wildlife habitat, conditions may be placed on planning approval to ensure that fauna are appropriately managed so that their welfare is not compromised. Management of wildlife associated with urban developments in Australian cities has typically involved mammals, with examples ranging from common brushtail and western ringtail possums [7,8,9], southern brown bandicoots [10], flying foxes (*Pteropus* spp.) [11] and eastern grey kangaroos [12]. Public concerns regarding lethal methods, such as culling, often influence the choice of management option [4].

The large size of kangaroos relative to most other mammals in urban/peri-urban environments, and the risk they pose due to vehicle collision, has led to public pressure to manage these populations appropriately [12,13,14,15,16]. Human–wildlife conflict is potentially greater for those larger urban mammals, and any management action is unlikely to go unnoticed [17]. Analogous to this situation is the management of deer populations, another large herbivore, in metropolitan areas of North America, where similar human-wildlife conflict issues apply [13]. Community consultation in relation to kangaroos has indicated that relocation rather than lethal removal is preferred [15,17], yet the long-term success and humaneness of relocating large numbers of kangaroos is not well understood [12,16]. A single study that monitored the outcomes of an eastern grey kangaroo (*Macropus giganteus*) relocation of just 10 individuals recorded an 80% survival rate after four months and 60% after one year [12]. 

A recent relocation of western grey kangaroos (*Macropus fuliginosus*) from an area subject to ongoing residential development on the periphery of Perth in Western Australia provided an opportunity to assess the survivorship and movement of these kangaroos post-release. The objective was to determine the outcome of relocating a relatively large number of individuals to inform future decisions regarding kangaroo management practices in urban areas. Specifically, we document survivorship, movement and activity patterns of the kangaroos over 12 months following relocation.

## 2. Materials and Methods 

### 2.1. Relocation

Western grey kangaroos were relocated from an urban development site (Paramount Estate) at the Perth outer suburb of Baldivis on the Swan Coastal Plain of Western Australia (32.3536° S, 115.8213° E). The development site (approximately 90 ha) was situated within an urban landscape with housing well established on the northern and western boundaries, and clearing for development had already taken place on the southern and eastern boundaries. Previous land use was for farming with the area having a grassy understorey and scattered tuart trees (*Eucalyptus gomphocephala*). A population of kangaroos remained isolated in the area. The relocation process (i.e., capture and transport to the release site) was undertaken by a contractor originally engaged by the property developer. A summary is provided below.

Prior to relocation, 154 kangaroos (52 with one pouch young) were herded into a large holding enclosure (approximately 4.2 ha) constructed of temporary fencing, by personnel on foot and all-terrain vehicles. They were then left undisturbed for two days to settle before relocation commenced. Some kangaroos escaped the enclosure and required re-herding at various times during the relocation. Before each relocation session (nine mornings in total), a sub-set of kangaroos were herded by personnel on-foot into a smaller fenced enclosure (approximately 0.05 ha) within the larger enclosure, where they were darted and sedated. Darting involved the use of a tranquilising gun (CO_2_ powered dart gun) and darts containing Zoletil^®^ 100, a mixture of tiletamine and zolazepam, at a dose rate of 5–10 mg/kg. Sedated kangaroos were measured, weighed, ear-tagged, a sub-set collared, placed in transport bags, and injected with diazepam when considered necessary by the on-site veterinarian. Any kangaroo that had suffered significant physical trauma as a result of hitting fences or other objects during herding and darting were euthanised either by, or under instruction from, the on-site veterinarian.

Kangaroos were transported to the relocation site in a covered trailer, laid out on a foam mattress on the trailer floor. At the relocation site, kangaroos were placed into a 0.06 ha soft release enclosure constructed of 1.8 m high panels of temporary fencing, internally lined with dark green shade cloth, to allow for recovery. Kangaroos that did not survive the journey (13) had their collars (if fitted) and ear-tags removed. Water was available ad libitum within the enclosure. After 24 h, kangaroos that were mobile were released. Those exhibiting severe signs of capture myopathy were euthanised. 

**Figure 1:**
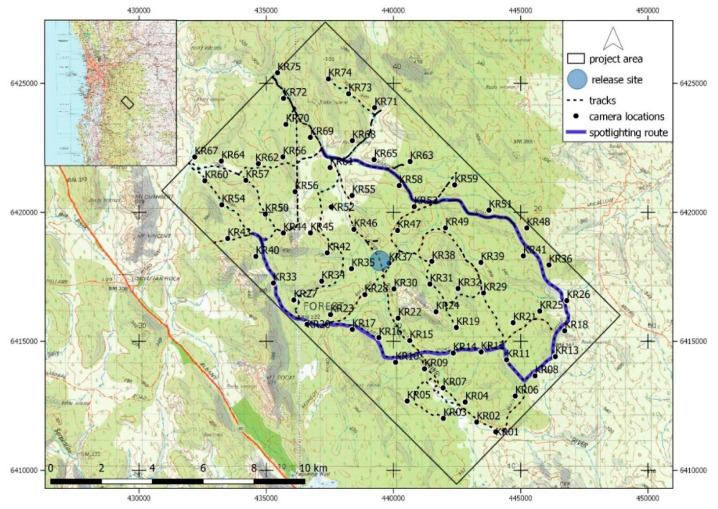
Relocation area showing point of release, camera trap array and driven spotlighting route.

A site in the Jarrahdale State Forest, 50 km to the east of Baldivis in the Darling Range (32.3723° S, 116.3565° E; Figure 1) was found to meet all the criteria for selection as a suitable relocation site. Calculations using known movements of kangaroos were made in relation to the proximity of private and agricultural areas, as well as distances to main roads to limit the likelihood of collisions with vehicles. There also needed to be continuity of habitat to allow the kangaroos to freely disperse. The release site was within an area of more than 1900 km^2^ of continuous forest with undulating topographic relief ranging from valley floors of 240 m above sea level to occasional hills as high as 550 m. There was a single lane highway 8 km to the south, agricultural land 12.5 km to the east and a mining operation 16 km to the south. Public access to the site required a permit as it was a quarantined plant disease risk area. There was also a reasonable network of minor tracks from which monitoring activities could be undertaken.

**Figure A1:**
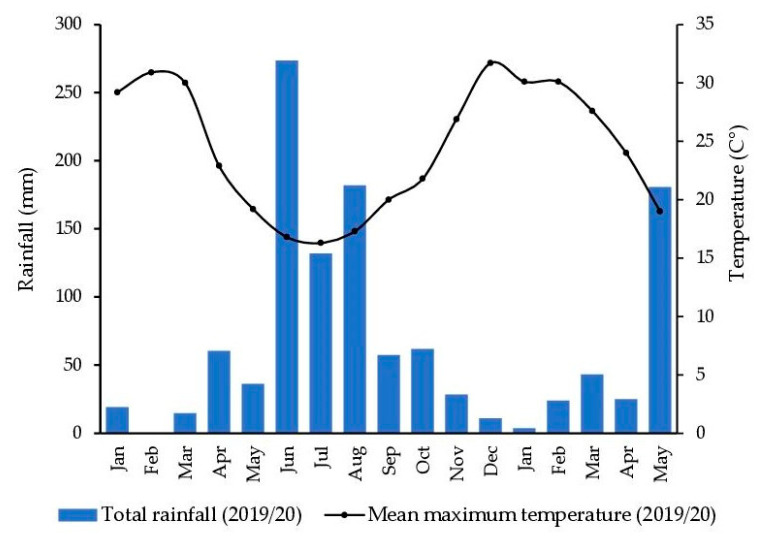
Total monthly rainfall (Bureau of Meteorology Jarrahdale weather station) and mean maximum monthly temperature for the period January 2019 to May 2020 (Bureau of Meteorology Karnet weather station) in the relocation area [62].

Vegetation at the relocation site consisted primarily of jarrah (*Eucalyptus marginata*) and wandoo (*E. wandoo*) open forest and had a mixture of fire ages ranging from recently burnt to areas that had been unburnt for more than six years. Prescribed burning was also not planned for the area. Although conditions were dry at the time of release (between 17 and 28 May 2019), there were at least two small dams holding water within a 5 km radius of the release site. There was also a water trough less than 1 km away, and water was made available at the relocation site. Annual rainfall for Jarrahdale in 2019 was 877 mm which was below the average of 1170 mm. The total rainfall for May was 36 mm which was well below the average of 153 mm, though the total in June was 274 mm which was above the average of 224 mm. Total monthly rainfall and mean maximum monthly temperature from January 2019 to May 2020 are provided in Appendix A Figure A1. The main difference between the development and relocation sites was that the former was primarily grassland with scattered trees, whereas the latter consisted of woodland. 

Pre-release assessment showed the area to already support western grey kangaroos and a smaller macropod, the brush wallaby (*Notamacropus irma*), although neither in large numbers. Spotlight surveys (see below) prior to the relocation recorded 0.17 western grey kangaroos per kilometre along a 30-km transect. No other faunal species likely to interact directly with the relocated kangaroos was known to occur in the study area.

### 2.2. GPS and VHF Telemetry

A range of Global Positioning System (GPS) telemetry devices from several manufacturers were investigated for bulk, weight, battery life, robustness, automated collar release mechanisms, remote programming, delivery time, cost and available collar sizing. Only one manufacturer was able to meet all the requirements and supply collars within the short lead time of six weeks. 

Two types of telemetry devices were selected to assess survivorship and monitor movement patterns of the kangaroos: (1) GPS collars (Sirtrack Pinnacle Pro Medium collars with satellite upload, solar assist and VHF beacon); and (2) Very High Frequency (VHF) collars (Sirtrack V6C 164C). GPS collars weighed between 440 and 460 g (<2.5% body weight of an adult western grey kangaroo) and had an internal collar circumference of 220–290 mm. They were also further modified by lining the inside of the collars with neoprene (5 mm thick self-adhesive) to improve the fit. Manufacturer fitted timed-release devices (TRD) were set to activate 365 days from when collars had been fitted.

As the priority for this study was to understand survivorship of a sub-set of relocated kangaroos over a 12-month period, it was important to balance frequency of positional fixes with frequency and packet size of data uploads to satellite to ensure endurance of the transmitters. Collars were programmed to take a fix every half hour for the first month while the kangaroos established themselves in their new environment, and every hour thereafter. The upload of data via satellite was after every eight positional fixes. All GPS collars were fitted with a small solar panel to extend battery life. 

VHF collars, which are lighter and have considerably less bulk than GPS collars, were used on smaller animals, including sub-adults, to assess survivorship rather than detailed movement patterns. These had an internal circumference of 160–300 mm and weighed 40–47 g (<0.3% body weight of a sub-adult western grey kangaroo). A weak link, designed to break over time, of 5 cm long, 1 cm wide and 1.5 mm thick rubber band (*n* = 5) or doubled over elastic of the same dimensions but 0.5 mm thick (*n* = 5) was inserted into each collar. This allowed for some expansion to accommodate the growth of sub-adults and removed the necessity of recapturing animals to remove collars as the rubber would perish over time. The rubber was attached at each end using two staples and contact adhesive, covered by heat shrink.

Mortality sensors were integrated in all collars and set to trigger after 12 h without motion. For VHF collars, the frequency of emitted pulse increased when in mortality mode, while for GPS collars, an email and text message was sent via satellite to a pre-determined address, as well as the VHF beacon pulse increasing in frequency. The VHF signal in the GPS collars was set to operate from 0800 to 1600 each day and was used to physically locate animals when necessary. Animals were located within 24 h of receiving a mortality signal which allowed for an assessment of the cause of death when retrieving the collar. 

A sample of 30 kangaroos (approximately 20% of the original estimated total population to be relocated), to be fitted with GPS collars, was considered sufficient to indicate survivorship and to provide detail on movement patterns of relocated adult kangaroos. The inclusion of a further ten animals with VHF collars would also improve the quality of statistical inference about survivorship while also sampling an age class that was too small for GPS collars. 

All collars were fitted to the kangaroos while they were sedated prior to transport to the relocation site. Sex, age class (adult, sub-adult, juvenile), reproductive status, neck circumference and weight, along with date and time of sedation for each kangaroo were recorded. The size of each animal was assessed, and a GPS or VHF collar was fitted to a sub-set to be relocated on each day. All kangaroos were also given a unique numbered ear-tag (Allflex two-piece minitag) while under sedation. Tags were coloured differently for each day of the relocation operation. Ear tags were positioned on the right ear for males and left ear for females, and reflective tape was also applied to both sides of the tag to facilitate identification of relocated animals. 

Each VHF-collared kangaroo was searched for daily over the first 14 days, then once a week for the next two months, then monthly for the remainder of the study. This was undertaken from a combination of vehicle and on foot using either a Sirtrack Ultra or Communication Specialist R1000 VHF receiver and Yagi three element antenna. The vehicle was also fitted with a VHF 3 dB mopole antenna tuned to 150.7 MHz—the centre of the frequency range of the VHF collars. On two occasions (15 August 2019 and 31 January 2020), an aircraft fitted with two Yagi antennae to the wing struts was used to locate some VHF-collared animals due to the difficulty in locating them on the ground. 

### 2.3. Relocated Kangaroos

Between 17 May and 28 May 2019, a total of 122 kangaroos (86 females and 36 males) was relocated to the Jarrahdale State Forest relocation site; 49% of the females were carrying small, unfurred pouch young. All kangaroos were captured in the early morning with temperatures not exceeding 25 °C. The number of individuals, sex and collar type fitted on each day are presented in Table 1.

GPS or VHF collars were fitted to a total of 67 animals; although there were only 40 collars (30 × GPS and 10 × VHF), several were re-used from deceased animals. The original intent was to distribute collars evenly between males and females as well as different size classes and across each of the relocation days. However, the high rate of mortality post-relocation (see below) meant that collars were fitted to almost every animal that fulfilled the size criteria (i.e., neck circumference ≥ 19 cm for GPS collars and ≥17 cm for VHF collars). As a result, 40 females and nine males were fitted with GPS collars, and nine females and nine males were fitted with VHF collars (i.e., 55% of relocated kangaroos). Size characteristics of GPS and VHF collared animals are given in Table 2.

### 2.4. Movement and Activity Patterns

Positional data were used to estimate overall dispersal and subsequent spatial and temporal activity patterns. These patterns were compared immediately after release, and during subsequent periods as the kangaroos adjusted to their new environment. Average and total daily movement data for all collared animals were calculated, although spatial patterns of VHF collared animals were not examined in any detail, as the primary aim for these individuals was to assess survival. Animals that died within 24 h of relocation were not included in the assessment of movement pattern. Where there were sufficient data, GPS fixes where animals remained largely resident (i.e., excluding linear movements of short duration) were used to estimate home range size based on both 95% minimum convex polygons and kernel density estimates. We applied these two estimators for comparative purposes as there is no universal standard and it also allows for comparison with other studies which have used either or both measures. Patterns of diel activity were also examined in relation to variation in distances moved throughout the day and across seasons.

### 2.5. Camera Traps and Spotlighting

To add to the information from collared kangaroos, the use of remote sensor camera traps and driven spotlight surveys to determine survivorship and movement of the relocated kangaroos without collars was examined. These techniques were also used to gain a better understanding of the distribution and relative abundance of resident western grey kangaroos at the relocation site. Recent studies have indicated that camera traps provide value in terms of documenting activity patterns and behaviour of eastern grey kangaroos—e.g., [18,19].

An array of 75 non-lured Reconyx HP2X Hyperfire camera traps was established across the relocation area using a constrained randomised design one month prior to the animals being relocated. Distance between cameras was at least 750 m and offset from tracks by at least 50 m. Camera separation distances of 750 m gave good coverage over the release area while ensuring a high probability of independence of detections between cameras over short timeframes. Offsetting cameras from roads was primarily for security reasons. Kangaroo detection reliability of the selected camera model was assessed by positioning two cameras side by side at 10% of the camera locations. This showed that there was minimal variability in detection rates of kangaroos between the two replicates, and collectively, the data were almost identical. The mean detection rate was 24.2 for both replicates (SD = 14.9 and 14.7, respectively). Cameras were operational for the duration of the study and checked every three months to change batteries and data cards. 

Spotlighting using two handheld spotlights (100-W Lightforce LFEF170CC) was undertaken from a vehicle along approximately 30 km of tracks around the periphery of the relocation site, over three consecutive days once a month for two months pre-release and repeated for the first two months post-release. Spotlighting was discontinued after this time due to a lack of sightings and low number of surviving relocated kangaroos. A transect was travelled in one direction on dusk and then the other direction in full darkness. The number of individuals sighted, the time they were sighted and geographic coordinates when sighted were recorded.

### 2.6. Data Analysis

Data analysis was undertaken using the R statistical programming environment [20] with “adehabitatHR” [21], “rgdal” [22], “rgeos” [23], “maptools” [24], “sp” [25] and “psych” [26] packages. Camera trap imagery was catalogued and managed using the open source database, Colorado Parks and Wildlife (CPW) Photo Warehouse [27]. The open source geographic information system Quantum GIS (QGIS) was used for spatial graphics [28]. 

Determining the accuracy of GPS positional fixes and filtering inaccurate data is essential for the analysis of animal movement [29]. This is often accomplished by using set cut off points for the dilution of precision values (DOP). However, the relationship between location error and DOP can be extremely weak [30], resulting in either rejection of accurate data or inclusion of inaccurate data. Novel approaches to filtering data by comparing altitude values derived from a digital elevation model (DEM) to those from the GPS device for the same location have been proposed [31]. This approach was used with ASTER (Advanced Spaceborne Thermal Emission and Reflection Radiometer) Version 3 Digital Elevation Model (DEM) data, excluding GPS point data that varied by more than 100 m from the DEM in elevation. This resulted in the rejection of 3655 data points from all GPS collars, or 8.9% of fixes.

### 2.7. Ethics Statement

Collaring and monitoring of kangaroos were approved under the Department of Biodiversity, Conservation and Attractions Animal Ethics Committee Approval Licence to Use Animals for Scientific Purposes No. 2019-06A and Regulation 17 Licence No. SC001489. The capture and transport of relocated kangaroos, as well as oversight of recovery from sedation, were operational activities undertaken by contractors.

## 3. Results

### 3.1. Survivorship

Of the 67 collared animals, 12 did not survive the first day of the relocation. On assessment by a veterinarian or experienced and qualified field zoologists, a further nine collared and five non-collared animals were euthanised between one and three days post-relocation due to severe stress. These individuals were either unable to stand or showed behaviours such as a wide stance to stay upright, were hunched over with their heads down and were continually drooling at the time of release from the enclosure and were not going to recover.

Of the 49 animals with GPS collars, only three survived for the full 12-month period: two females and one male. Of the 18 VHF collared animals, just three survived longer than 85 days, and they were known to have been alive for 85, 205 and 266 days, after which the collars had broken away and they were no longer tracked. 

For collared kangaroos that died, survival time was relatively short with only seven animals surviving longer than 50 days (4 VHF and 3 GPS). Excluding the animals that died within the first day after relocation and the three GPS animals that were alive at the end of the project, the average duration of survival was 9.5 days (*n* = 46, SD = 14.6). 

**Figure 2:**
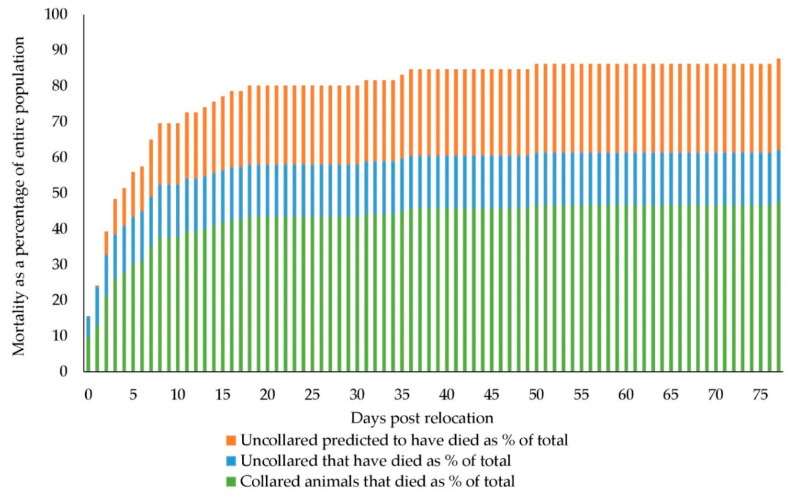
Mortality rate of western grey kangaroos as a percentage of the total number relocated.

The proportion of collared animals known to have died throughout the project (i.e., 88%) applied to the entire relocated population of 122 predicted that 107 kangaroos did not survive. The mortality rate of the relocated kangaroos in respect to days post-relocation is shown in Figure 2. The steepest part of the curve was over the first three days, with 47 animals (32 collared and 15 uncollared) not surviving, and a further 12 predicted to have died based on the proportion of known deaths, representing 48% of relocated animals. Mortality continued at a rapid rate through to day eight post-relocation, with 46 of the collared and 18 non-collared animals deceased and about 21 others predicted to have died (70% of relocated animals). By day 18, over 80% of the animals relocated were predicted not to have survived. From this point onwards, attrition was at a much slower rate, with a further five deaths of collared animals occurring, and three others predicted to have died up until the last recorded death on day 77.

The deaths of four individuals were classified as misadventure with one confirmed to have been hit by a vehicle and another thought to have suffered the same fate. Two others were thought to have been killed by illegal hunting. Three of these were collared, and an ear tag was recovered for the fourth. These four were not used in mortality calculation rates but when added to the known and predicted mortality, the number believed to have died totalled 111 or 91% of the relocated animals. 

Samples of thigh muscle, heart muscle, kidney and liver were collected from the last six GPS collared animals that died. These samples came from four females and two males that had survived from 15 to 77 days post-relocation. These were submitted to a veterinary pathologist at the Western Australian Department of Primary Industries and Regional Development to diagnose the cause of death. From field examination, these individuals appeared to have died quite suddenly, with no apparent prolonged immobility or struggle and all had full stomachs from feeding. Of the six animals assessed, the majority (80%) had severe congestion of glomeruli and interstitial vessels in the cortex and medulla of the kidney, 40% showed evidence of necrosis of skeletal muscle and one individual had liver necrosis, but no conclusive cause of death was possible.

### 3.2. Movement Patterns

**Figure 3:**
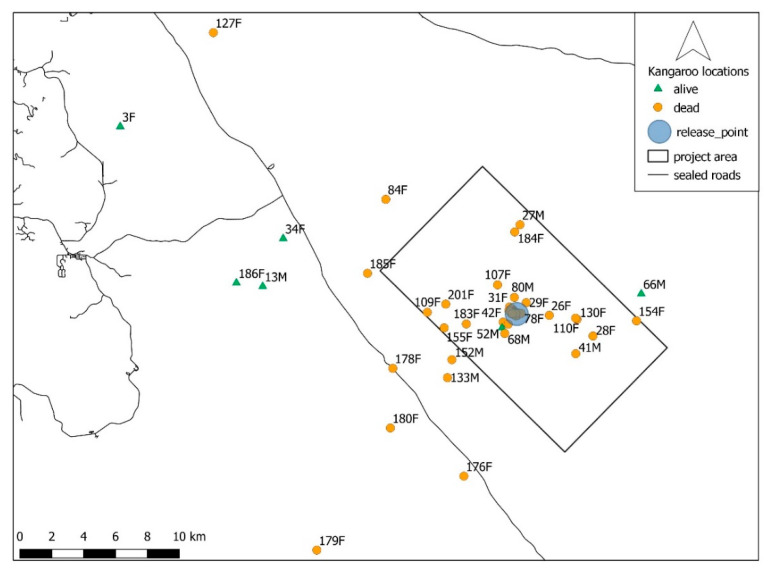
Maximum distance moved by relocated western grey kangaroos from the release site. Triangles represent those animals surviving for the duration of the study. Circles represent those animals that died.

**Figure A2:**
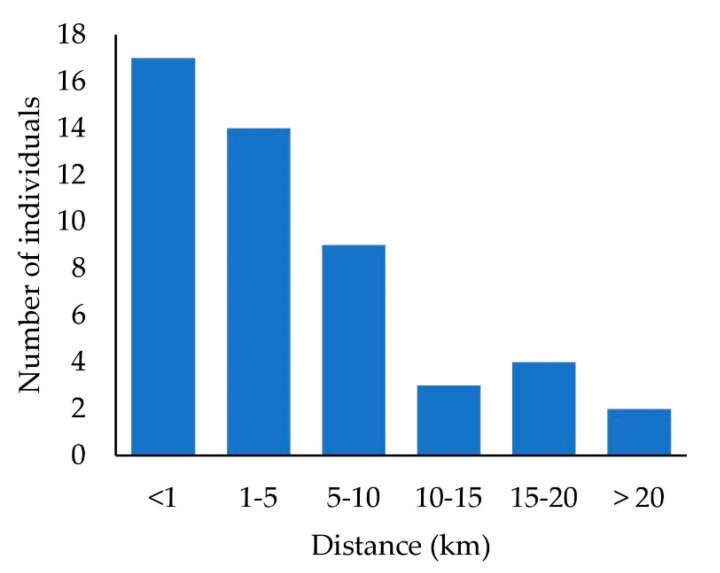
Maximum distances western grey kangaroos moved from the release site following relocation.

The maximum linear distance that any animal travelled from their point of release was 27.8 km with a mean of 5.4 km (Figure 3). For VHF collared animals, the maximum linear distance moved was 26 km with a mean of 5.2 km. Average and total daily movement data are shown in Appendix A Table A1 for all GPS collared animals. The number of animals and distribution of distances moved from the relocation site is shown in Appendix A Figure A2.

**Figure A3:**
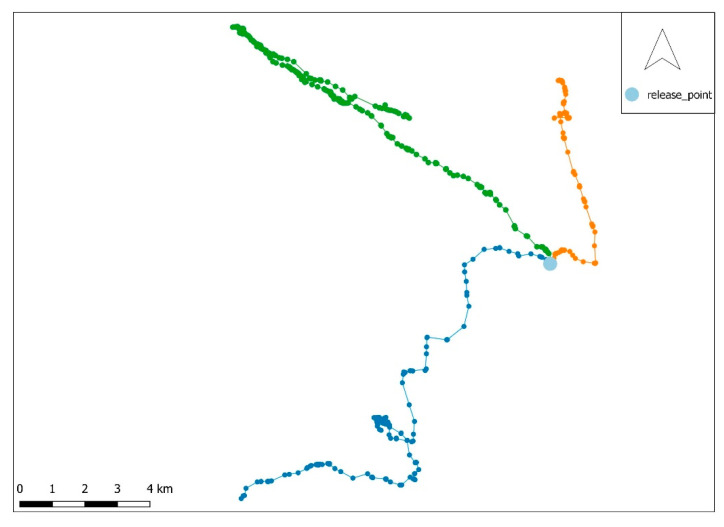
Post-release movements of three western grey kangaroos that survived >5 days (half-hourly intervals) but died during study.

GPS-collared animals that survived for less than 5 days (*n* = 18) remained near to the release site, and only half of them made movements of 100 m or more between half-hourly fixes. Animals that survived beyond five days but died during the study period (*n* = 13) tended to make movements in the one general direction and rarely returned to a prior location (Appendix A Figure A3). Their movement pattern exhibited some differences to that of animals that survived the study period. They spent an average of 76% (SD = 9.2) of their time moving less than 40 m between half-hourly fixes (classified here as resting or feeding), 12% (SD = 3.7) of their time making short movements of 40–100 m between half-hourly fixes and 12% (SD = 7.5) of their time making larger movements of over 100 m between half-hourly fixes. The three animals that survived the duration of the study initially spent 86% (SD = 3.2) of their time resting or feeding, 9% (SD = 2.0) in short movements and just 5% (SD = 2.5) in larger movements.

**Figure 4:**
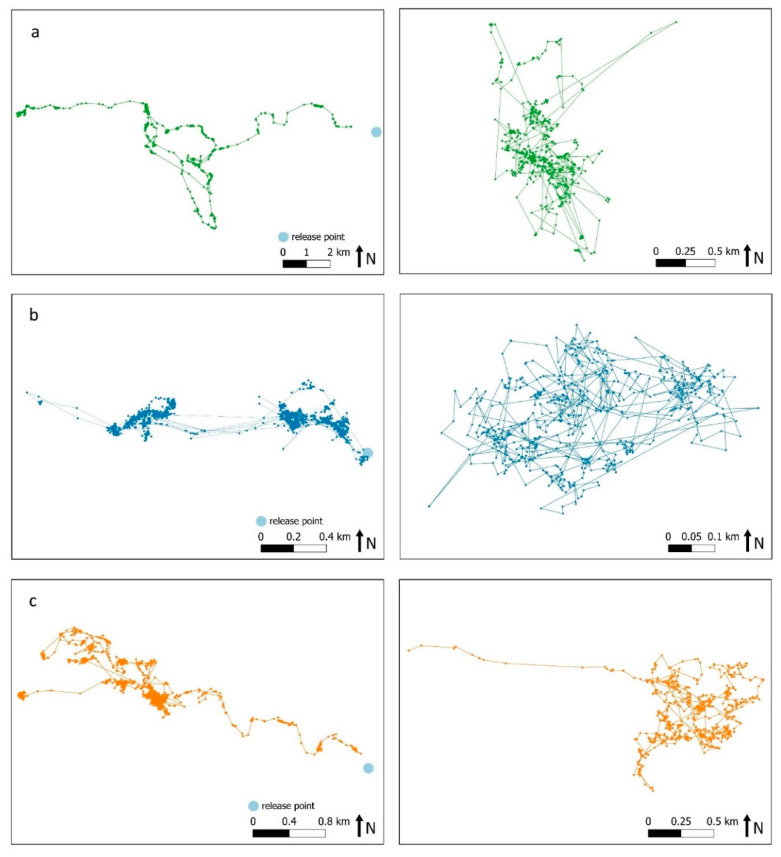
Movement of the three surviving individuals (**a**) 3F, (**b**) 186F and (**c**) 13M during the first month post-release using half-hour intervals (left) and during the 12th month post-release using one-hour intervals (right).

**Figure A4:**
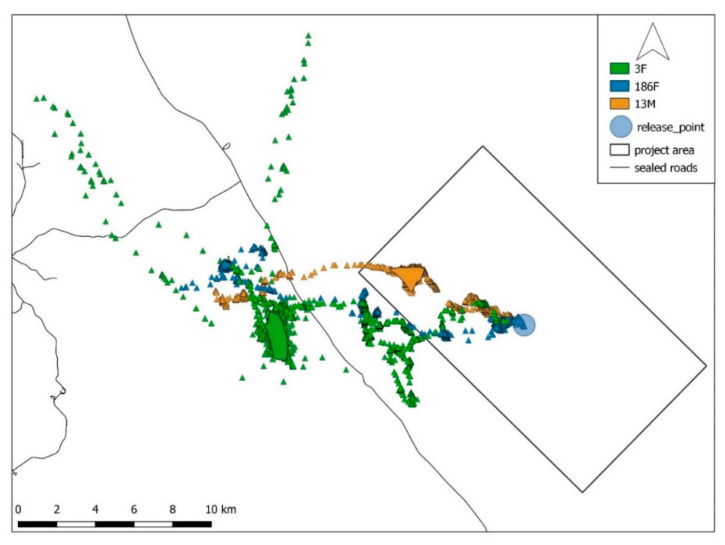
Movement of surviving GPS collared western grey kangaroos (triangles) and the primary areas they occupied (polygons) following relocation.

Quantitative spatial analysis was only possible for three GPS collared animals (13M, 3F, 186F), as the movement patterns of most individuals, along with a generally short survival time, prevented the calculation of stable home ranges. During the first month post-release, these three animals regularly revisited apparent points of focus (Figure 4). As GPS fixes were reduced to one-hourly after one month, their hourly movements were re-examined for the first month post-release. During this time, 78.7% (SD = 5.2) of movements were less than 40 m, 12.7% (SD = 4.0) were 40–100 m and 8.6% (SD = 3.7) were greater than 100 m. Disturbance due to unforeseen mining exploration in the general area appeared to influence the movement patterns of one kangaroo with increased daily movements observed. By 12 months, all three kangaroos had developed more discrete home ranges. Most movements occurred between regular points of focus (Figure 4), and a greater proportion of time was spent undertaking movements of 40 to 100 m (20%, SD = 1.7) and movements of over 100 m (11%, SD = 4.1). There were occasions when they made lengthy movements, travelling far from the release site and outside the project area (Appendix A Figure A4), but always returned to the same general area. 

**Figure 5:**
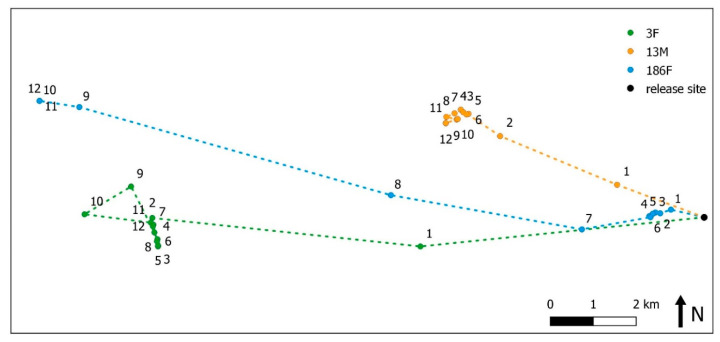
Geometric centre of area occupied for each month post-release for individual kangaroos 13M, 186F and 3F.

**Figure A5:**
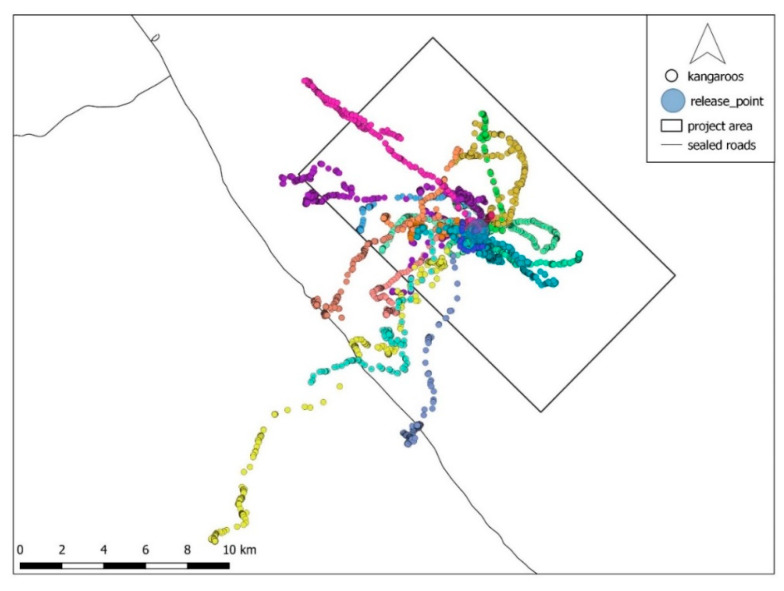
Movement of GPS collared western grey kangaroos (coloured circles) that died following relocation.

The geometric centre of area occupied for the three GPS collared kangaroos above in relation to months post-release is shown in Figure 5. Both 3F and 13M immediately departed the release site taking between ~30 and 45 days, respectively, to establish a relatively stable area of occupancy, where they remained for the next 12 months. The second female, 186F, remained close to the release site for the first six months post-release, moving no further than 1.5 km. Over the following three months, she progressively moved around 15 km to the west and occupied the same small area for the last three months of monitoring. Using monthly GPS data where they were resident and still remained at the end of 12 months, excluding linear movements of short duration outside these areas, the mean 95% minimum convex polygon was 8 (SD = 2), 33 (SD = 6) and 25 ha (SD = 5) for 186F, 3F and 13M, respectively. Using the same data for 95% kernel density estimates, mean values were 10 (SD = 3), 50 (SD = 9) and 35 ha (SD = 6), respectively. Home range polygons and movement data for the 12-month study period are shown in Appendix A Figure A4. Movement patterns for all other GPS collared animals are given in Appendix A Figure A5.

**Figure 6:**
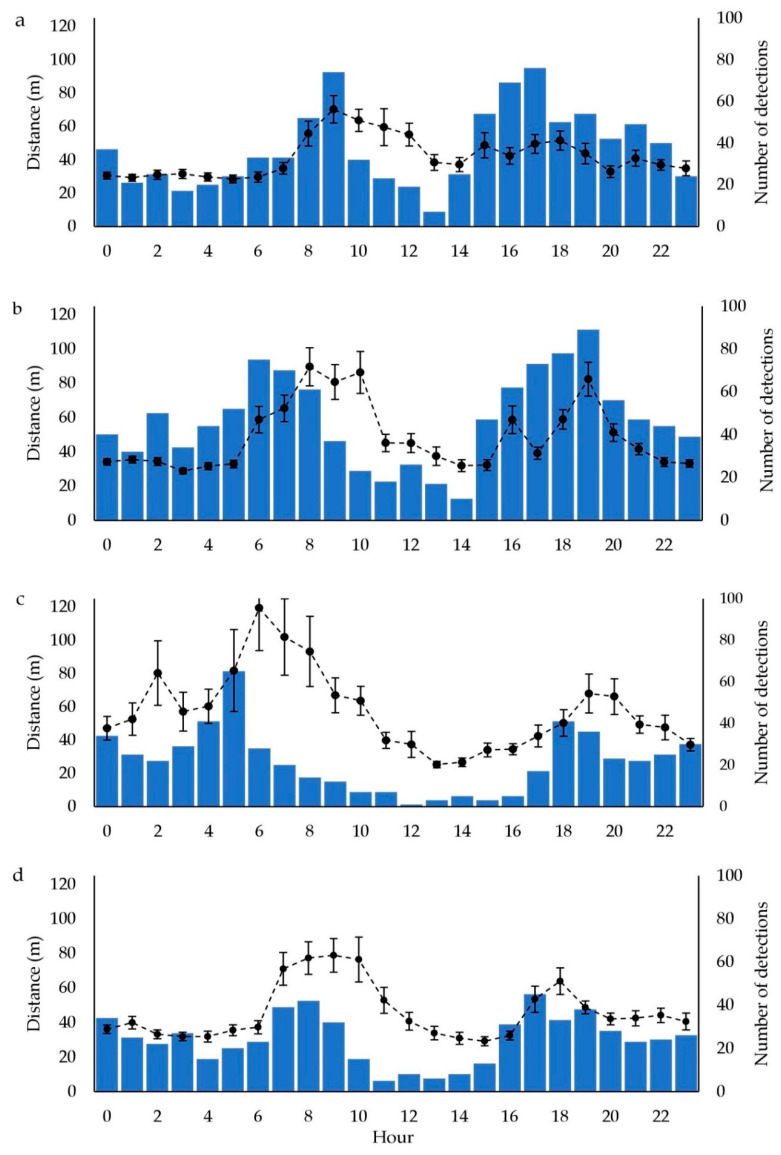
Average total distance moved per hour for the kangaroos surviving for the duration of the project (line plot with standard error bars and left axis) and number of single detections of resident kangaroos for each camera within each hour from the camera array (bars and right axis) for (**a**) winter, (**b**) spring, (**c**) summer and (**d**) autumn.

Patterns of average diel activity for the three long term surviving GPS collared animals showed variation in distances moved throughout the day and across seasons (Figure 6). Peak daily activity, as determined by maximum mean distances moved, occurred during the morning with a second but smaller peak occurring in the late afternoon or early evening. Activity in the summer months began earlier than it did in the cooler months starting at around 0600 but was closer to 0900 in the winter. The afternoon period of activity in summer was also about an hour later than it was for other seasons with a peak at around 1900. While mean maximum distance moved ranged from a low of 70 m (SD = 123) in winter to a high of 92 m (SD = 285) in summer, there was variability in these data with maximum movements in peak hours of up to 1.8 km in winter and 3.1 km in summer. 

### 3.3. Camera Monitoring

Just 31 collared animals remained within the area of the camera grid or spotlighting transects; 28 of these survived for less than a month (X̄ = 6.1 days, SD = 6.6), two survived for 36 and 50 days, respectively, and only one survived for the duration of the project.

While the primary objective of the camera trap array was to monitor the distribution and detection rates of relocated kangaroos, the rapid demise of most relocated kangaroos meant that there were too few animals to contribute data for that purpose. Only three relocated kangaroos were detected, and these were all within 14 days post-relocation. The low number of detections of marked kangaroos also supports the mortality predictions above for those uncollared animals.

The diel activity of the GPS collared kangaroos indicated patterns of daily and seasonal variability. We examined camera detection data to assess whether there was a level of concordance in broad patterns of activity for GPS collared kangaroos and resident kangaroos. Data from cameras were available for 346 consecutive days beginning on the 24 April 2019. Independent detections were defined as a single detection of a western grey kangaroo during any hour for any camera. This resulted in a total of 3119 independent detections of western grey kangaroos and a mean daily detection rate of 9.0 (SD = 4.7). 

For the resident kangaroos, the diel assessment of winter months indicated that peak activity was later in the morning (8% at 0900) and earlier in the afternoon/evening (8.5% at 1700) than it was during summer months (12% at 0500 and 8% at 1800). Data for spring and autumn indicate peak activity earlier in the morning than for winter but later than for summer (6.7% at 0600 and 7.2% at 0800, respectively). Peak activity in the afternoon was later than for winter but earlier than summer. Peaks were slightly higher in the afternoons/evenings for winter, spring and autumn but much higher in the morning during summer. These same general trends were broadly reflected in the data from GPS collared animals (Figure 6).

All 75 camera trap sites were occupied by resident kangaroos at some stage throughout the duration of the project with mean detection rates per camera of 39.2 (SD = 35.3, range 5 to 222). Fifteen percent of sites had detections for every month of the project, 50% of sites had detections for at least ten or more months and 90% of sites had detections for at least six or more months. 

### 3.4. Spotlight Monitoring

No relocated kangaroos were observed during spotlighting surveys, though the first post-release survey was 30 days after the relocation was completed, by which time 80% of the kangaroos were thought to have died. An average of 0.2, 0.3 and 0.4 resident western grey kangaroos per km of transect was detected across the three nights sampled in April/May, July and September, respectively. Most sightings were of solitary animals; however, occasionally, groups of up to five individuals were sighted.

## 4. Discussion

Management of kangaroos at the urban interface can be challenging with limited management options available [12,32,33]. In terms of managing population size, lethal control is by far the most widespread management practice, but non-lethal options such as reproductive control, relocation and employing the use of deterrents are commonly suggested as alternatives, particularly in urban areas [12,33,34].

Impending housing development of the site on which a population of western grey kangaroos had already been isolated by urban expansion was the reason behind the relocation of these kangaroos. While there is a perception by the community that relocation is a more humane option for minimising anthropogenic impacts on imperilled wildlife, as the outcomes of these fauna relocations are often poorly documented, evidence to support this belief is largely lacking [1,4]. 

### 4.1. Post-Release Survival

While this study is the first detailed assessment of a large scale western grey kangaroo relocation in Western Australia, the high mortality rate reported here differs markedly from other studies that necessitated the capture of kangaroos. For example, Coulson et al. [13] sedated, captured and marked 360 eastern grey kangaroos on a golf course and did not record any deaths, although habituation to human presence may have helped here. A large-scale sterilisation program in western Sydney of 5825 eastern grey and red kangaroo captures over a 13-year period, resulted in 523 deaths (9%), 19 of which were attributed to capture myopathy [35]. On the smaller-scale, Munn et al. [36] reported no losses of 11 western grey kangaroos taken from the wild and relocated to an enclosure. Chachelle et al. [37] likewise documented no deaths resulting from the sedation and capture of 20 western grey kangaroos. Higginbottom and Page [12] documented the relocation of 10 eastern grey kangaroos and reported 80% surviving after four months and 60% surviving for a year. Note that not all these studies involved transportation of the kangaroos.

That most kangaroos succumbed so soon after relocation suggests that these deaths were primarily related to stress associated with capture [38]. Capture myopathy, a condition associated with animal capture, restraint (chemical or physical) and transport, is reportedly common in macropods [39,40,41,42] and is the likely cause of death for many of the kangaroos. The impact of repeated exposure to stressors, such as herding, capture, handling and transportation is also known to be additive [38]. A recent review by Breed et al. [43] indicates that capture myopathy, globally, accounts for a significant number of deaths associated with translocation, with the condition also prevalent in a wide range of African, North American and European ungulates.

Excessive stress in animals often results in overexertion causing metabolic imbalance leading to, among other issues, skeletal or cardiac muscle damage and necrosis [43,44]. Once this damage occurs, the prognosis for recovery is extremely poor. The syndrome presents with a variety of clinical signs including anxiety, unsteady or stiff gait, muscle tremors, shivering, muscle stiffness, muscular weakness, bent neck, partial paralysis, increased respiratory rate or lethargy [39,40,43,44]. This condition can be acute, resulting in death within hours to days, but may also manifest sometime later, with death occurring several weeks or even over a month after the initial trauma [39,43]. Kangaroos that died rapidly post-relocation or were euthanised in this study generally presented with clinical signs indicative of capture myopathy. While there was no definitive cause of death for kangaroos that died weeks after the relocation, it is possible that they also suffered a protracted manifestation of capture myopathy. Their behaviour and movement patterns were somewhat unlike those kangaroos that survived for the duration of the study (see [38]).

The best approach to managing risks of capture myopathy is to minimise stress to the animals as much as possible. Breed et al. [43] suggest that the rate of capture myopathy can be indicative of how well animal welfare was considered. Vogelnest and Portas [42] advise capture and restraint techniques must be carefully planned and executed by experienced and skilled operators who are familiar with macropod behaviour and restraint techniques. Every aspect of a relocation process from initial capture through restraint, transportation and recovery should be conducted with the critical objective of minimising stress [38,43]. 

### 4.2. Release Location and Timing

While capture myopathy is likely to be the primary factor for the failure of this relocation, there are other potential contributing factors that should be considered. Here, the forest location where the kangaroos were released differed from the relatively open grassy area from which they came, and potentially competing conspecifics were resident in the relocation area. It was intended that a camera array would provide some insights into distribution and integration of marked relocated animals with resident kangaroos (i.e., detection ratios of marked and unmarked animals); however, with so few relocated animals surviving, this was not possible.

Natal experience is important to habitat-selection by dispersing animals [45] and animals released to novel environments may not respond appropriately to unfamiliar cues regarding resources, even if a sufficient amount is present [46]. This may be further exacerbated by stress-induced impairment of learning and memory [38]. However, alternatives to forested areas for large relocations of kangaroos within the general vicinity of a metropolitan landscape are likely to be few [12]. Open grassed areas are usually associated with agricultural land use, and without sympathetic landholders, are not an alternative. Even then, substitutes that could support large numbers of relocated animals and enable unrestricted movement would be extremely limited. In this relocation, the presence of resident western grey kangaroos in the release location demonstrated that the area was suitable for this species.

While public access was prohibited without a permit in the release location, there were encounters with illegal hunters, firewood collectors and off-road motorbike riders, and at least two kangaroos were likely to have succumbed to unlawful hunting. The long-distance movements of some kangaroos post-release (> 10 km) resulted in a further two deaths from vehicle impacts on a section of highway eight kilometres away. This distance moved was greater than that generally reported for wild populations of western grey kangaroos [47,48] although Priddel [49] reported movements of greater than 30 km for three individuals (however, median distance moved was 3 km). The extent to which human activity may disturb and add additional stress to relocated animals is difficult to gauge [38], although behavioural observations during the study suggested that they were sensitive. For example, disturbance associated with mining exploration in the vicinity of one of the long-term surviving animals coincided with increased daily movements of this kangaroo over several weeks. A similar study documenting post-release survival of white-tailed deer (*Odocoileus virginianus*) relocated from a metropolitan area to a rural setting in Illinois, North America, reported that capture-related stress, accidents with vehicles and losses to hunters were the major factors that led to lower survival of the deer [50].

In many cases, timing of relocation is not always solely determined with the welfare of animals in mind but driven more by economics, logistics and even convenience [1]. For macropods, ethical issues around relocations are increased when there is the potential for young to be ejected from the pouch or for dependent young to be separated from their mother [51]. Fortunately, at the time of this relocation, most mature females had pouch young in early development, and there were no young at heel considered to be dependent.

Although conditions were generally dry for some weeks following relocation, water was available from at least three sources (dams and an artificial watering point). Full stomachs of the autopsied kangaroos also indicate that they were finding sufficient food resources. So, it is unlikely that lack of food and water resources contributed to the relocation failure. Likewise, daily temperatures during the relocation did not exceed that recommended by Breed et al. [43]. 

### 4.3. Movement Patterns 

Exploratory behaviour is vital to the successful establishment of animals in a novel environment [52,53,54,55]. Out of all the GPS-collared animals, just the three kangaroos that survived for the duration of the study exhibited organised exploratory behaviour i.e., movements away from their initial release location that would allow them to gain knowledge of their novel environment and build experience in locations of high value. Such behaviour is the mechanism by which animals learn of resources, potential dangers and social opportunities so that they might optimally exploit their environment [53]. Over time, the movement patterns of these three animals became more regular and contained focal points that were repeatedly revisited—behavioural patterns indicating that they were adapting well to their new environment [53]. The home range size estimates for these three kangaroos was generally smaller than reported in other studies of the same species (e.g., [37,47]).

Most other kangaroos remained near the release location and subsequently died there, or moved away in a series of unidirectional movements, without establishing any particular points that were revisited or formed centroids of exploratory behaviour. These erratic movements of the kangaroos that died relatively early in the study are suggestive of highly stressed individuals. A level of release-site fidelity is important to establishing new populations as it minimises deleterious dispersal [56,57,58,59,60]. One of the reasons implicated in the failure of relocations is extensive movements away from the unfamiliar release location [4,5,6], reducing the fitness of relocated animals in the early post-release stage [53]. 

Diel activity showed surviving kangaroos to be bimodal, moving greater distances between fixes in the morning periods than the afternoon or evening. This timing is similar to those found for eastern grey kangaroos by Henderson et al. [14]. Seasonal shifts in these patterns were evident with activity occurring earlier in summer than for other seasons and extending further into daylight hours during winter. Activity patterns of these relocated animals were broadly synchronised with camera detection rates of resident kangaroos in the same area, also suggesting behavioural stability. 

### 4.4. Post-Release Monitoring Approach

Due to the low survival rate of collared kangaroos, the value of GPS telemetry for monitoring movement patterns, and hence assessing adaptation to a new environment, was not fully realised during this study. Other studies using this technology have reported on the benefits it provides, particularly for tracking long-distance movements [14,61]. If the primary aim is to determine survival rates only, VHF collars are likely to be a more cost-effective option. In this study, the fate of all VHF-collared kangaroos was able to be determined. Even with aircraft tracking flights on two occasions, the cost per VHF-collared individual was only a fraction of the GPS collars (AUD$517 per collar compared to AUD$3115). 

Although the GPS collars were large and bulky, there did not appear to be any significant long-term welfare issues. Several animals that had GPS collars attached initially showed signs of distress when recovering from sedation, but it was unclear if it was related to the collar, a reaction to the drugs or a combination of both, as macropods can have prolonged and violent recoveries after being sedated with Zoletil^®^ [35]. However, the reaction appeared to dissipate quickly once the animals had recovered from sedation. No animals were displaying adverse reactions to collars when released from the enclosure. Photographic evidence of the three surviving GPS collared animals showed them to be wearing the collars well with no sign of rubbing or irritation during the wearing of the collar, or after the collar’s release. 

The GPS collars functioned well and the battery life lasted the required 12 months when collars remained on animals. There were some minor issues in that mortality mode did not always engage for the VHF signal even though a message that the animal was deceased had been received. Issues with the timed-release device (TRD) were more significant with one failing to release and a two-week delay in the release of another. Failure to release and delays in activation of a TRD have implications for animal welfare and collar recovery, particularly if working in remote and difficult to access areas, and potentially needing to recapture a wary animal. This constitutes a 33% failure rate—significantly greater than the already high 19% failure rate reported by Matthews et al. [61]. Signal transmission and battery life of the VHF collars also worked well, but there were some issues with mortality signals. Two failed to enter mortality mode when the animal had died, and one switched from mortality mode to live mode without movement of the collar. The retrofitted weak links built into these collars worked as planned with all releasing.

While the cameras were not effective for the intended purpose of this study, they did show that there was a relatively widespread and stable population of resident western grey kangaroos across the relocation area. Spotlighting also proved ineffective but further trials could test if detecting marked individuals by this technique can be achieved, and therefore, provide useful information.

## 5. Conclusions

Effective management of kangaroos at the urban interface is challenging. Here, the survival rate of western grey kangaroos was poor, with an estimated 80% of the 122 kangaroos perishing within the first month of relocation and only six collared kangaroos surviving for up to 12 months. This poor outcome raises concerns around the viability of relocating a relatively large number of kangaroos humanely. Based on the results of their study, Higginbottom and Page [12] considered that relocations should not be a wide-spread solution to the impact of development on kangaroos, as there are unlikely to be many sites that meet relocation criteria, and the stress associated with the capture and relocation often results in the death of animals. Stress associated with the relocation process was implicated as the cause of the early deaths of the kangaroos. This was supported by the short duration of survival of the majority of kangaroos, that a considerable proportion died in transit or required euthanising, those that survived for less than 5 days remained in close proximity to the release site and macropods in general are known to be highly susceptible to capture myopathy. As such, where relocation is the preferred course of action, it is essential to carefully consider the welfare of the animals, particularly in the capture and transport phase, and also follow up with appropriate methods to monitor and report on the success of the relocation [5,43,62,63].

## Figures and Tables

**Table 1:** Number of individual western grey kangaroos, sex and collar type (GPS: Global Positioning System or VHF: Very High Frequency) fitted on each day of relocation.

Date	Number Captured			GPS		VHF		Total Collared
	Male	Female	Total	Male	Female	Male	Female	
17 May 2019	2	14	16	2	7	0	1	10
20 May 2019	7	11	18	2	7	1	3	13
21 May 2019	12	8	20	1	3	4	0	8
22 May 2019	5	17	22	1	5	2	1	9
23 May 2019	4	9	13	0	3	2	1	6
25 May 2019	4	10	14	2	2	0	2	6
26 May 2019	2	5	7	1	3	0	1	5
27 May 2019	0	11	11	0	9	0	0	9
28 May 2019	0	1	1	0	1	0	0	1
TOTAL	36	86	122	9	40	9	9	67

**Table 2:** Number and size characteristics of GPS and VHF collared western grey kangaroos (S.D. = standard deviation).

Collar Type	Neck Circumference			Weight			Number
	Range (cm)	Mean (cm)	S.D.	Range (kg)	Mean (kg)	S.D.	
**GPS collar**							
Male	21–28	24.7	2.5	23.2–66.9	44.1	15.7	9
Female	19–24	21.3	1.1	18.7–31.4	25.2	3.5	40
**VHF collar**							
Male	17–22	19.7	1.7	15.5–25.5	20.1	3.1	9
Female	18–20	19.0	0.7	16.3–23.5	19.1	2.1	9

## Appendix A

**Table A1:** Average daily maximum linear distance and average total daily distance moved by each GPS collared relocated western grey kangaroo (#: total number of days monitored).

Individual	# days	Maximum Linear Distance			Total Distance		
		Mean (km)	Maximum (km)	S.D.	Mean (km)	Maximum (km)	S.D.
3F	370	0.68	10.17	1.03	1.43	11.52	1.28
4F	2	0.05	0.10	0.06	0.24	0.47	0.32
5F	2	0.16	0.31	0.21	0.45	0.88	0.61
13M	383	0.30	5.04	0.47	0.71	5.34	0.52
27M	4	1.41	2.33	1.11	2.29	4.16	1.93
28F	9	0.74	1.89	0.63	1.33	2.52	0.82
29F	5	0.18	0.39	0.17	0.54	0.86	0.38
30F	2	0.21	0.41	0.29	0.47	0.94	0.67
31F	8	0.27	0.45	0.19	1.41	2.53	0.85
41M	36	0.60	3.97	0.80	1.23	4.67	0.94
42F	2	0.77	1.54	1.09	1.41	2.81	1.98
43F	2	0.05	0.09	0.06	0.33	0.66	0.47
63F	2	0.10	0.19	0.13	0.25	0.49	0.34
78F	3	0.18	0.28	0.15	0.85	1.54	0.78
79F	2	0.06	0.10	0.06	0.30	0.87	0.49
84F	12	1.28	2.64	1.03	1.77	3.26	1.06
94F	3	0.04	0.07	0.03	0.42	0.67	0.36
102F	6	0.13	0.19	0.06	0.61	1.20	0.38
107F	4	0.54	1.49	0.67	1.63	3.37	1.53
109F	35	0.31	3.09	0.56	0.92	3.65	0.59
130F	15	0.83	2.69	0.82	1.39	2.95	0.86
133M	6	1.09	3.94	1.42	1.69	4.33	1.52
152M	7	1.20	4.21	1.35	2.43	5.82	2.15
155F	4	1.06	1.92	0.68	1.38	2.29	0.87
156F	2	0.06	0.10	0.05	0.38	0.73	0.50
176F	18	1.44	9.94	2.80	2.76	20.76	5.02
178F	14	0.80	3.53	1.05	1.43	4.22	1.16
179F	13	1.65	5.02	1.59	3.02	9.22	2.81
180F	6	1.70	5.06	1.68	2.99	7.41	2.56
181F	2	0.13	0.17	0.06	0.46	0.84	0.53
183F	3	1.33	3.47	1.87	3.71	8.89	4.56
184F	11	1.85	4.07	1.37	3.68	8.00	2.69
185F	77	0.32	2.04	0.41	0.82	2.41	0.48
186F	363	0.28	4.90	0.49	0.74	7.09	0.69
201F	14	1.47	4.25	1.44	3.36	8.45	2.79
